# Commentary On: Three Calcium Hydroxylapatite–Based Dermal Fillers Marketed in Mexico: Comparison of Particle Size and Shape Using Electron Microscopy

**DOI:** 10.1111/jocd.70340

**Published:** 2025-07-29

**Authors:** Nabil Fakih‐Gomez, Jonathan Kadouch, Cristina Muñoz‐Gonzalez

**Affiliations:** ^1^ Department of Facial Plastic and Cranio‐Maxillo‐Facial Surgery Fakih Hospital Khaizaran Lebanon; ^2^ Practice for Aesthetic Dermatology ReSculpt Clinic Amsterdam the Netherlands

**Keywords:** aesthetic medicine, collagen remodeling, dermal filler

We read with great interest the article titled “Three Calcium Hydrofxylapatite‐Based Dermal Fillers Marketed in Mexico: Comparison of Particle Size and Shape Using Electron Microscopy” by Gilberto A. Sánchez Rico and Silvia Beatriz Andrade Canto, recently published in the *Journal of Cosmetic Dermatology*, 2015;24(3):e70100. doi: 10.1111/jocd.70100. While we appreciate the effort to explore calcium hydroxylapatite (CaHA) particle morphology, we would like to respectfully express several scientific concerns regarding the methodology and conclusions drawn.

First, the authors' decision to avoid washing and centrifugation—standard steps for isolating CaHA particles—raises questions about data validity. They claim to have avoided centrifugation to prevent microsphere fragmentation; however, previously validated protocols have demonstrated that Radiesse (Merz Aesthetics, Frankfurt, Germany) microspheres remain morphologically intact following multiple high‐speed centrifugation cycles (5000 rpm for 5 min, repeated five times), with no evidence of damage [1]. Moreover, studies such as those by de Moraes Nobre et al. and Kunzler et al. successfully performed centrifugation (up to 10 000 rpm) without microsphere fragmentation [1, 2]. In contrast, scanning electron microscopy (SEM) images of HarmonyCa (Allergan Aesthetics, Irvine, CA, USA) microspheres showed broken, irregular, and fragmented microspheres, with polymer deposits in the fissures [1].

Second, the analysis presented in the article is not representative. Only one image per filler and one particle per image were used for morphological assessment, despite millions of microspheres being present in each syringe. In contrast, other studies quantified 150+ particles per sample to ensure statistical robustness. This selective imaging raises the possibility of cherry‐picking favorable data. Further, the authors fail to disclose their conflicts of interest, with the first author directly reporting that he is a trainer and speaker for Allergan Medical Institute on social media. Therefore, the lack of transparent disclosure and potential bias toward Allergan may have resulted in selective image analysis with bulk SEM imaging.

Third, the claim that HarmonyCa microspheres are “uniform and spherical” is inconsistent with the authors' own micrographs. Figure 1 (500×) and Figure 2 (1000×) clearly show fractured, irregular particles, contradicting the assertion in Figure 3's caption. Prior studies using SEM have also identified broken, hollow, and anisomorphic HarmonyCa particles, including variability in surface texture.

**FIGURE 1:**
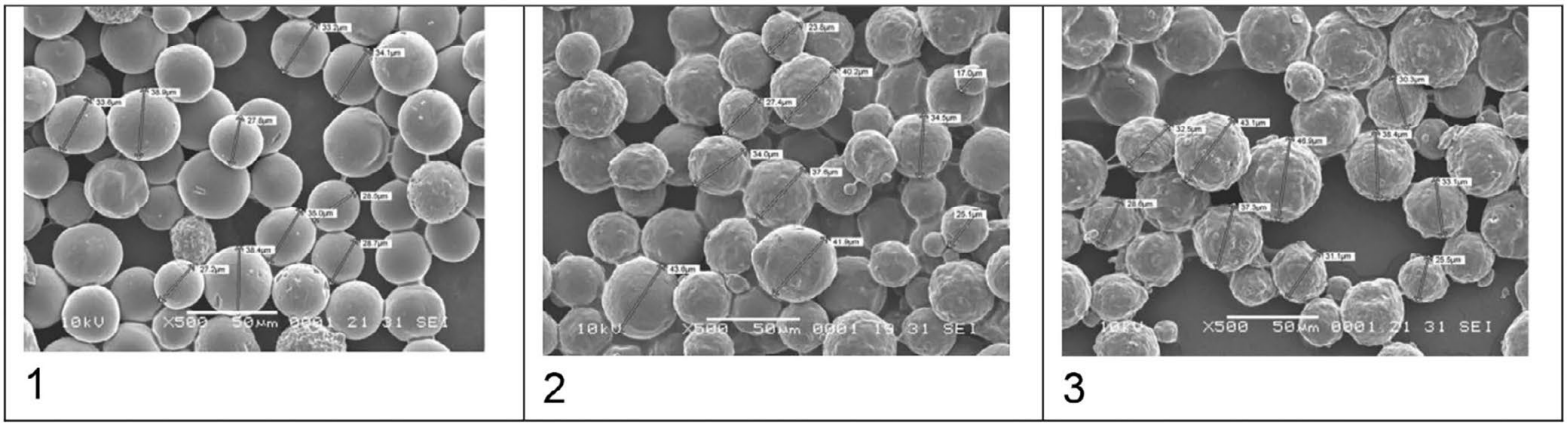
Comparative SEM microphotographs showing HArmonyCA (1), Radiesse (2), and Hydroxyfill (3) ×500. Figure 1 from published article: Sanchez Rico GA, Canto SBA. Three Calcium Hydroxylapatite‐Based Dermal Fillers Marketed in Mexico: Comparison of Particle Size and Shape Using Electron Microscopy. *J Cosmet Dermatol*. 2025;24(3):e70100. doi: 10.1111/jocd.70100. PMID: 40035469; PMCID: PMC11877991.

**FIGURE 2:**
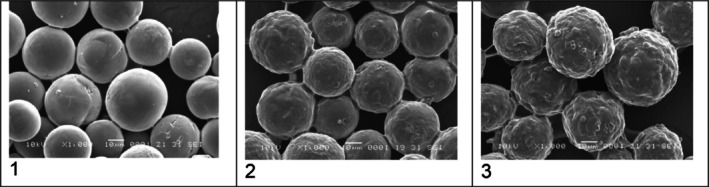
Comparative SEM microphotographs showing surface morphology in HArmonyCA (1), Radiesse (2), and Hydroxyfill (3) ×1000 magnification. Figure 2 from published article: Sanchez Rico GA, Canto SBA. Three Calcium Hydroxylapatite‐Based Dermal Fillers Marketed in Mexico: Comparison of Particle Size and Shape Using Electron Microscopy. *J Cosmet Dermatol*. 2025;24(3):e70100. doi: 10.1111/jocd.70100. PMID: 40035469; PMCID: PMC11877991.

**FIGURE 3:**
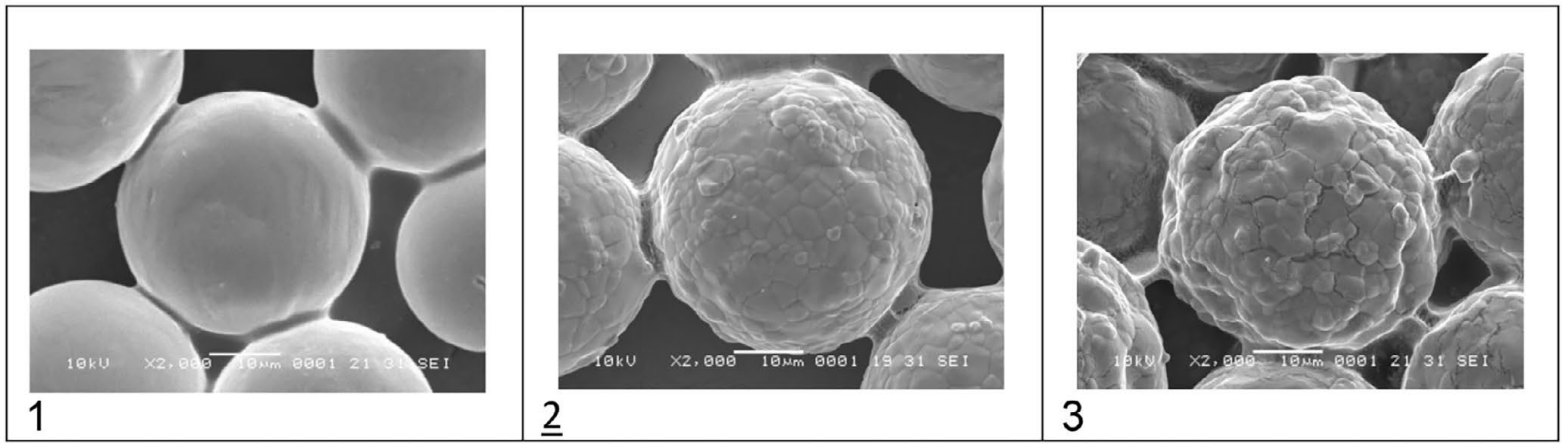
Comparative microphotographs at 2000× of HArmonyCA showing an even surface and spherical shape (1), Radiesse showing a slightly rough surface (2), and (3) Hydroxyfill showing a rougher surface and a less homogeneous shape. Figure 3 from published article: Sanchez Rico GA, Canto SBA. Three Calcium Hydroxylapatite‐Based Dermal Fillers Marketed in Mexico: Comparison of Particle Size and Shape Using Electron Microscopy. *J Cosmet Dermatol*. 2025;24(3):e70100. doi: 10.1111/jocd.70100. PMID: 40035469; PMCID: PMC11877991.

Fourth, the lack of a discussion section precludes scientific interpretation. The authors fail to reconcile their findings with a large body of literature showing that Radiesse microspheres are uniformly smooth and spherical, with limited phagocytosable material [1, 3, 4]. By omitting comparative discussion or methodological limitations, the study misses an opportunity for constructive scientific dialogue.

Fifth, the authors express vague concerns about Radiesse's biocompatibility, yet this contradicts published evidence. Multiple studies have shown Radiesse to be immunologically inert, producing limited macrophage activation and lower phagocytosable content relative to PLLA and other CaHA‐based materials [5, 6]. Histological studies support its role in collagen I and III stimulation, fibroblast activation, and low granuloma risk.

Sixth, the quantitative analysis appears flawed. The graphs comparing particle sizes include ≤ 10 particles per filler—insufficient for any statistically meaningful conclusion, especially given the natural variability across batches and syringe sites. We support the effort to better characterize dermal fillers, but accurate, reproducible, and statistically sound methods are essential. We encourage future studies to align with standardized SEM protocols, use representative sampling, and transparently report limitations. We appreciate the opportunity to share these comments and hope they contribute constructively to the scientific discourse in the field of cosmetic dermatology.

## Ethics Statement

The authors have nothing to report.

## Consent

The authors have nothing to report.

## Conflicts of Interest

The authors N.F.‐G., J.K, C.M.‐G. are consultants for Merz Aesthetics (Frankfurt, Germany).

## Data Availability

The authors have nothing to report.
